# Aphid symbionts and endogenous resistance traits mediate competition between rival parasitoids

**DOI:** 10.1371/journal.pone.0180729

**Published:** 2017-07-10

**Authors:** Laura J. Kraft, James Kopco, Jason P. Harmon, Kerry M. Oliver

**Affiliations:** 1 Department of Entomology, University of Georgia, Athens, Georgia, United States of America; 2 Department of Entomology, North Dakota State University, Fargo, North Dakota, United States of America; Institut Sophia Agrobiotech, FRANCE

## Abstract

Insects use endogenous mechanisms and infection with protective symbionts to thwart attacks from natural enemies. Defenses that target specific enemies, however, potentially mediate competition between rivals and thereby impact community composition. Following its introduction to North America to control pea aphids (*Acyrthosiphon pisum*), the parasitoid *Aphidius ervi* competitively displaced other parasitoids, except for the native *Praon pequodorum*. The pea aphid exhibits tremendous clonal variation in resistance to *A*. *ervi*, primarily through infection with the heritable bacterial symbiont *Hamiltonella defensa*, although some symbiont-free aphid genotypes encode endogenous resistance. Interestingly, *H*. *defensa* strains and aphid genotypes that protect against *A*. *ervi*, provide no protection against the closely related, *P*. *pequodorum*. Given the specificity of aphid defenses, we hypothesized that aphid resistance traits may contribute to the continued persistence of *P*. *pequodorum*. We conducted multiparasitism assays to determine whether aphid resistance traits mediate internal competition between these two solitary parasitoid species, but found this was not the case; *P*. *pequodorum* was the successful internal competitor across lines varying in susceptibility to *A*. *ervi*. Next, to determine whether resistance traits influence competitive interactions resulting in the stable persistence of *P*. *pequodorum*, we established replicated cages varying in the proportion of resistant aphids and recorded successful parasitism for each wasp species over time. As expected, *A*. *ervi* outcompeted *P*. *pequodorum* in cages containing only susceptible aphids. However, *P*. *pequodorum* not only persisted, but was the superior competitor in populations containing any proportion (20–100%) of resistant aphids (20–100%). Smaller scale, better replicated competition cage studies corroborated this finding, and no-competition and behavioral assays provide insight into the processes mediating competition. Genetic variation, including that acquired via infection with protective symbionts, may provide a supply of hosts susceptible only to particular enemies, mediating competition with effects on community richness and stability.

## Introduction

Diverse eukaryotic taxa show substantial variation in susceptibility to attack by specific natural enemies [1–5]. Most insect species, for example, are attacked by parasitoids, which typically kill their host before completing development, resulting in strong selection for hosts with resistance traits [6, 7]. Resistance to attack, however, can be costly, often leading to variation among individuals within a species [8–10]. Host resistance to parasitoids can be endogenous or mediated by infection with microbial symbionts, and each type of resistance may be specialized to particular natural enemies [11–17]. Variation and specialization in resistance to particular enemies may affect competitive interactions among parasitoid species [2, 18] and ultimately influence the species richness and evenness of the parasitoid community attacking the host.

Aphids (Hemiptera: Sternorrhyncha), a diverse group of phloem-feeding herbivores, often show tremendous variation in resistance to parasitoids that can arise from endogenous sources and infection with maternally transmitted bacterial symbionts [4, 13, 16, 19–27]. For example, the pea aphid, *Acyrthosiphon pisum*, is a leading model of protective symbiosis. In addition to the obligate, nutrient-provisioning symbiont, *Buchnera aphidicola* allowing exploitation of plant phloem [28], individual pea aphids can be infected with at least one of seven common heritable facultative symbionts (HFS) and each species is known or suspected to contribute to protecting the aphid host ([23] but see [29]). For example, several HFS species, including *Regiella insecticola*, provide protection against the entomopathogenic fungi *Pandora neoaphidis* [30–32] and *Hamiltonella defensa* defends against attacks by the hymenopteran parasitoid *Aphidius ervi* [33]. Infection with *H*. *defensa* confers varying levels of protection against attack by *A*. *ervi* depending on symbiont strain and associated bacteriophages called APSEs that infect the bacterial symbiont [21, 22, 34, 35]. For example, aphids infected with *H*. *defensa* and phage variant APSE3 receive high levels of protection, while those infected with strains lacking APSE receive no protection [21]. Infection with *H*. *defensa* can be costly, and balancing selection likely contributes to the maintenance of intermediate frequencies observed in field populations [9, 36, 37]. While most clones lacking infection with *H*. *defensa* in lab studies have been found to be highly susceptible to attack by *A*. *ervi*, some aphid clones lacking HFS do have endogenous resistance [13].

The pea aphid was accidentally introduced to North America in the 19^th^ century, and numerous native and introduced parasitoids successfully attacked this aphid until the introduction of *A*. *ervi* in 1959 [38]. After its introduction, however, *A*. *ervi* (Braconidae: Aphidiinae) emerged as the dominant parasitoid of pea aphids, outcompeting and displacing other native and introduced parasitoid species. The native wasp, *Praon pequodorum*, also an aphidiine braconid, was the only parasitoid not displaced by *A*. *ervi* in field populations, although the proportion of pea aphids successfully parasitized by this wasp dropped sharply (ca. 40% to < 10%). Prior studies have identified processes that may contribute to the continued persistence of *P*. *pequodorum*. First, *P*. *pequodorum* may be a superior internal competitor (i.e. within host) in instances of multiparasitism with *A*. *ervi* and related aphidiine braconids, where individuals of both species compete within a single aphid host, but only one can survive [39–41]. Second, *A*. *ervi* is the superior external (i.e. among hosts) competitor, exhibiting better foraging skills and faster attack rates relative to *P*. *pequodorum* [38], although *A*. *ervi’s* foraging efficiency is more negatively impacted by non-target aphids [42]. A recent study also found that diverse *H*. *defensa* strains and HFS-free innately resistant clones provide protection that is specific to *A*. *ervi* and has no significant effect on *P*. *pequodorum* development [12]. Hence, another possibility is that aphid resistance traits, both endogenous and symbiont-based may also contribute to the persistence of *P*. *pequodorum*. Resistance traits specific to *A*. *ervi* may result in a reservoir of hosts susceptible only to *P*. *pequodorum*, or directly impact internal competition during instances of multiparasitism. A recent study using a European pea aphid line found that *H*. *defensa* influenced internal competition between *A*. *ervi* and the distantly related parasitoid, *Aphelinus abdominalis* (Chalcidoidea: Aphelinidae) [18].

Here we examine whether the specificity of pea aphid resistance traits identified in our earlier study [12] influences competition between rival parasitoid species. First, we performed an assay to determine whether *H*. *defensa* or aphid-based defenses mediate internal competition between these two wasps during instances of multiparasitism. Second, we conducted population cage studies, at two scales, to determine whether resistance traits influence competitive interactions resulting in the stable persistence of *P*. *pequodorum*. Replicated cage treatments varied in the proportion of aphids carrying highly protective *H*. *defensa* (treatments ranged from 0% to 100%) or with 100% aphid-based resistance (large-scale assays only). We also conducted assays without competition, as well as behavioral assays, aimed at better understanding processes mediating competition.

## Materials and methods

### Aphid collection and rearing

Three experimental pea aphid lines were used in this study. Line AS3-Hd+ (collected from alfalfa, *Medicago sativa*, in Utah, USA 2007) is infected with an APSE3 containing *H*. *defensa* strain that confers high levels of resistance to *A*. *ervi*. Line AS30-Hd- resulted from the antibiotic curing [43] of AS3-Hd+ and thus shares the same aphid genotype, which is highly susceptible to *A*. *ervi* [13]. Line CJ1130-R (Utah alfalfa; 2012) is free of HFS but the aphid genotype is itself highly resistant to *A*. *ervi* [13]. Aphids were acquired from Utah State University research plots and did not require specific permission to collect nor were any protected or endangered species present in these fields. All aphid lines were established from single, parthenogenetic females, and were maintained in redundant cultures on fava plants (*Vicia faba*) in cup cages (Georgia (GA) experiments) or 60x60x60cm Bug Dorm 6610 cages (North Dakota (ND) experiments) reared at 20±1°C with a 16L: 8D photoperiod. Aphid lines were screened for expected symbionts as well as all other pea aphid HFS using the PCR primers and reaction conditions as in [37]. Across all lines, only *H*. *defensa* was detected, and this symbiont was only present in line AS3-Hd+, as expected. We further confirmed the presence of phage APSE3 using diagnostic PCR [21]. Aphid clonal lines were verified using microsatellite analysis prior to the experiments as in [13].

### Parasitoid collection and rearing

The *P*. *pequodorum* culture was established from field-collected mummies collected from North Dakota alfalfa. The GA and ND colonies were maintained by parasitizing cohorts of ~200 susceptible aphids in a pint-sized cage using 20 wasps over a period of ~10 hours. Parasitized aphids were then placed in a larger cage to mummify and held at approximately 21°C on 16L: 8D photoperiod. The GA *A*. *ervi* culture was established from wasps derived from several sources, including a commercial insectary (Rincon Vitova) and field-collected mummies from North Dakota and Wisconsin, while the ND culture contained only ND collected wasps. The *A*. *ervi* colony was maintained under similar conditions and on the same aphid lines as *P*. *pequodorum*.

### Do host resistance traits mediate internal competition during multiparasitism?

#### Experiment 1

A multiparasitism assay was conducted to determine whether aphid resistance traits affect internal competition between the two parasitoid wasps. Using same-aged, mated females of each *P*. *pequodorum* and *A*. *ervi*, we parasitized cohorts of 20 (X 10 replicates) third instar aphids that were A) highly susceptible (line AS30-Hd-), B) have symbiont-based resistance (AS3-Hd+), or C) have aphid-based resistance (CJ1130-R). Half the replicates (N = 5) for each treatment were parasitized first by *P*. *pequodorum* then again <6 hrs later with *A*. *ervi*, and half vice-versa; ensuring that females of each species lay only one egg in each aphid. There was no difference in multiparasitism outcome due to wasp parasitism order, (also seen in [41]), so we combined these for a single analysis per treatment. This study also reported that the time between parasitism events (4h to 24h) had little effect on outcome so we chose 6h to simplify logistics. Multiparasitized aphids were placed in cohorts of 20 on individual fava plants and maintained for 10 days or until mummification was visible to determine the competition outcomes.

### Can host resistance traits mediate competition between rival parasitoids?

#### Experiment 2

Population cages were established by placing 60 adult aphids of a particular treatment (20 aphids on each of three *Vicia faba* plants) in each cage (Bug Dorm_1 DP1000 30x30x30cm). For Experiment 2A, Treatments were A) uninfected, susceptible aphids (line AS30-Hd-), B) aphids infected with APSE3-*H*. *defensa* which is highly resistant to *A*. *ervi* (line AS3-Hd+), and C) uninfected aphids with a resistant aphid genotype (line CJ1130-R). For Experiment 2B, treatments were A) 50% with symbiont-based resistance (AS3-Hd+) and 50% highly susceptible aphids (AS30-Hd-), B) 80% with symbiont-based resistance (AS3-Hd+) and 20% with highly susceptible aphids (AS30-Hd-), and C) 20% with symbiont-based resistance (AS3-Hd+) and 80% highly susceptible aphids (AS30-Hd-). Four replicate cages of each treatment (A- C) were created for a total of 12 cages. The 60 adult aphids used to seed each cage were allowed to reproduce for four days producing a cohort of approximately 800–1100 2^nd^ and 3^rd^ instar nymphs, the instars preferred for both *A*. *ervi* and *P*. *pequodurum* suitable for parasitism [44–46]. At this point, we introduced 10 mated female wasps of each *A*. *ervi* and *P*. *pequodorum* to each cage and allowed them to parasitize aphids for 24 hours before they were aspirated from cages. After ~10 days, we removed all mummies and recorded the total number of each parasitoid species per cage. Mummies are easily distinguished as *P*. *pequodorum* pupates underneath the aphid’s mummified exoskeletons and *A*. *ervi* within it. Mummies from each species were placed in separate cages to eclose as adults and mate before being used in the next round of parasitism. Population cages were reset with 20 aphid adults per plant and given 3–4 days to reproduce as above to maintain a static resistance target. This is to ensure similar number of aphids among treatments as susceptible aphids suffered high mortality due to parasitism. After such time, all wasps that emerged from a specific replicate cage were placed back into the same cage and again allowed to parasitize aphids for 24 hours. The cages were repeated until either a) one wasp species lost, b) enemy frequencies stabilize, or c) four bouts of parasitism occurred.

#### Experiment 3

We also conducted smaller scale competition assays to complement our larger cage studies but with much greater replication, and to determine if the presence of a competitor affected the pattern of parasitism of the parasitoids relative to our no competition experiment (below). Treatments varied by cohort composition, and included either 100% AS3-Hd+ aphids, 100% AS30-Hd- aphids, or 50% of each. Each 40x10cm cylindrical replicate cage contained a cohort of 30 aphids on a 10-day-old fava seedling. Next, we simultaneously introduced single 2–3d-old, mated *A*. *ervi* and *P*. *pequodorum* females into each cage and allowed both wasps to forage for 4 hrs (10:00 to 14:00). To determine rates of successful parasitism, we then counted the number of mummies produced by each parasitoid species after 10 days. Parasitoids were provided with honey and water prior to experiments, and had never before encountered aphids. The number of aphids and exposure time were chosen to reduce the likelihood of superparasitism and larval competition and thus make the parasitism assay more reflective of wasp foraging behavior. Each treatment was replicated 24 times, although four replicates (2 from 100% AS3-Hd+ and 2 from 50% of each) were not included in our analysis because one or both of the parasitoids were not found at the end of the oviposition period.

#### Experiment 4

We also performed parasitism assays in the absence of competition to determine patterns of parasitism of each wasp species in response to different proportions of resistant and susceptible aphids. Treatments were the same as above: either 100% AS3-Hd+ aphids, 100% AS30-Hd- aphids, or 50% of each. In this assay, each cohort of 20 aphids was exposed to single, mated female of either species, *A*. *ervi* or *P*. *pequodorum*, for 4 hrs (10:00 to 14:00), with mummies counted after 10 days. This created a 3x2 treatment factorial, with the first factor being the three aphid treatments and the second the two parasitoid species. The experiment was replicated over four experimental blocks, each spaced one week apart. The first two experimental blocks included 8 replicates of each treatment, while the latter two experimental blocks included 12 replicates of each treatment. In a small number of replicates, the parasitoid could not be found at the end of the 4 hr oviposition period to be removed. Because the fate of these parasitoids is unknown, and they may have had the opportunity to continue ovipositing after the 4 hr oviposition period, these replications were excluded from analysis. Similarly, replicates in which no mummies formed were excluded from analysis to use consistent methodology with [33].

#### Experiment 5: Parasitoid behavior

Because our parasitism assays indicated that the parasitism success of *A*. *ervi* changes in response to both aphid resistance traits and the presence of *P*. *pequodorum*, we conducted behavioral observations to see if the foraging behavior of *A*. *ervi* changes with these factors. We set up a 3x2 factorial arrangement of treatments. The first factor was the composition of the aphid cohort exposed to the parasitoids: 100% AS3-Hd+, 100% AS30-Hd-, or 50% of each. The second factor consisted of whether the *A*. *ervi* foraged alone or foraged alongside a *P*. *pequodorum*. The arenas consisted of 10-day-old fava seedlings infested with a total of 30 second instar aphids. We used 2–3 day old female parasitoids that were provisioned with honey and water and that had never encountered aphids prior to the experiments.

We recorded the amount of time parasitoids spent walking, resting, grooming, and feeding on honeydew or from extrafloral nectaries, in addition to encounters with aphids and encounters with other parasitoids. For each aphid/parasitoid encounter, we recorded whether the aphid was parasitized, the aphid escaped (e.g. the parasitoid thrust with her ovipositor, but the aphid dropped, backed away, or kicked and was not parasitized), or the aphid was rejected (e.g. the parasitoid antennated the aphid, but did not attempt to oviposit). These observations focused on whether or not the parasitoid probed the aphid with her ovipositor, but we did not confirm whether or not eggs were laid. However, the decision to lay eggs and the number of eggs laid may vary according to host type [47]. On several occasions we noted *P*. *pequodorum* approaching *A*. *ervi*. Upon making contact, the *P*. *pequodorum* would behave evasively (e.g. orient away from *A*. *ervi* or fly away), behave aggressively (lunge toward *A*. *ervi* and, in one instance, seize the *A*. *ervi* with the front legs and stab with the ovipositor), or ignore the *A*. *ervi* (continue walking at a constant pace with no more than a 45^o^ change in direction). In case these interactions affected parasitism, they were also analyzed for differences by aphid cohort.

To reduce the number of inactive parasitoids, we initially tested each parasitoid in a 10 cm Petri dish with 20 susceptible second instar aphids. Parasitoids that did not attack an aphid in the Petri dish within five minutes were discarded and not used in experiments. Wasps that did parasitize an aphid were introduced to the cage containing the aphids and fava seedling and their behaviors were recorded for one hour. Observations were terminated early if no parasitoids made contact with an aphid for 15 minutes. Host plants were small enough that even when two parasitoids were included, both could be simultaneously observed. Each treatment was replicated 10 times except for the 50% of each aphid line without *P*. *pequodorum* treatment, which was replicated 11 times.

### Statistical analyses

In the population cages, each mummy count from each cage at each new time point was compared against the values in the T0 time point through a Fisher’s Exact Test to determine whether the wasp proportions significantly differed from starting wasp proportions. For the multiparasitism assay, a logistic regression analysis was used to compare *P*. *pequodorum* success among lines and thereby determine whether aphid resistance mediated internal competition. A Fisher’s Exact Test was also used with the multiparasitism assays to determine whether competition outcome differed from the null expected (i.e. equal internal competitive ability). For the smaller scale competitive and non-competitive parasitism assays, we used ANOVA to test for differences in the number of mummies produced by each wasp species separately to test for block effects in the non-competitive parasitism assays and to confirm that parasitism differed between the two pure aphid cohort treatments. For the smaller scale competitive and non-competitive parasitism assays, we used ANOVA to analyze differences in mummy production between the different aphid cohort compositions. Both wasp species were included in each ANOVA. To conduct statistical analysis, the number of mummies was square-root transformed to achieve normally distributed data. For the parasitoid behaviors, we used MANOVA to compare the time each parasitoid allocated to walking, grooming, resting, and feeding. We also used MANOVA to compare the number of aphids parasitoids encountered and the numbers of those encounters that included stings, aphid escapes, or rejections of aphids. We used MANOVA to compare the numbers of interactions between *A*. *ervi* and *P*. *pequodorum* in which *A*. *ervi* fled from, ignored, or attacked *P*. *pequodorum*. Finally, we compared the numbers of mummies that formed after each behavioral observation using ANOVA. All significant results in ANOVA were further resolved using Tukey’s HSD post-hoc tests. Statistical analyses were conducted in R v. 3.2.0.

## Results

### Aphid resistance traits do not mediate internal competition between rival wasps

A multiparasitism assay (Experiment 1) was conducted to determine whether symbiont- and aphid-based resistance mediates internal competition between *P*. *pequodorum* and *A*. *ervi*. We found no difference in outcome whether *A*. *ervi* or *P*. *pequodorum* attacked the aphid first thus results were combined (P = 0.3853). We find that *P*. *pequodorum* produces significantly more mummies than *A*. *ervi* in all three aphid lines (Table 1) indicating *P*. *pequodorum* is the superior internal competitor. We also found no significant differences among lines indicating that neither symbiont nor aphid-based resistance traits mediate multiparasitism outcomes between these two wasps (Table 1).

**Table 1:** Mean values (± SE) for surviving aphids, *P*. *pequodorum* mummies, *A*. *ervi* mummies, and dual mortality (both aphid and wasp died) for each aphid line. P value determined using Fisher’s Exact Test comparing total *P*. *pequodorum* mummies to total *A*. *ervi* mummies by cage. *Logistic regression analysis indicated **no significant difference** between this line and the susceptible control. (AS3-Hd+, P = 0.9964; CJ1130-R, P = 0.9968).

Aphid Line	Surviving Aphids	*P*. *pequodorum* mummies	*A*. *ervi* mummies	Dual Mortality	P value
AS30-Hd-	1.38±0.35	9.63±0.66	0.75±0.23	8.25±0.61	P<0.0001
AS3-Hd+ *	0.88±0.37	10.63±0.88	0.38±0.17	8.13±1.12	P<0.0001
CJ1130-R *	0.75±0.22	10.75±0.54	1.00±0.35	8.25±0.41	P = 0.0001

### Aphid resistance traits do mediate external parasitoid competition

**Fig 1:**
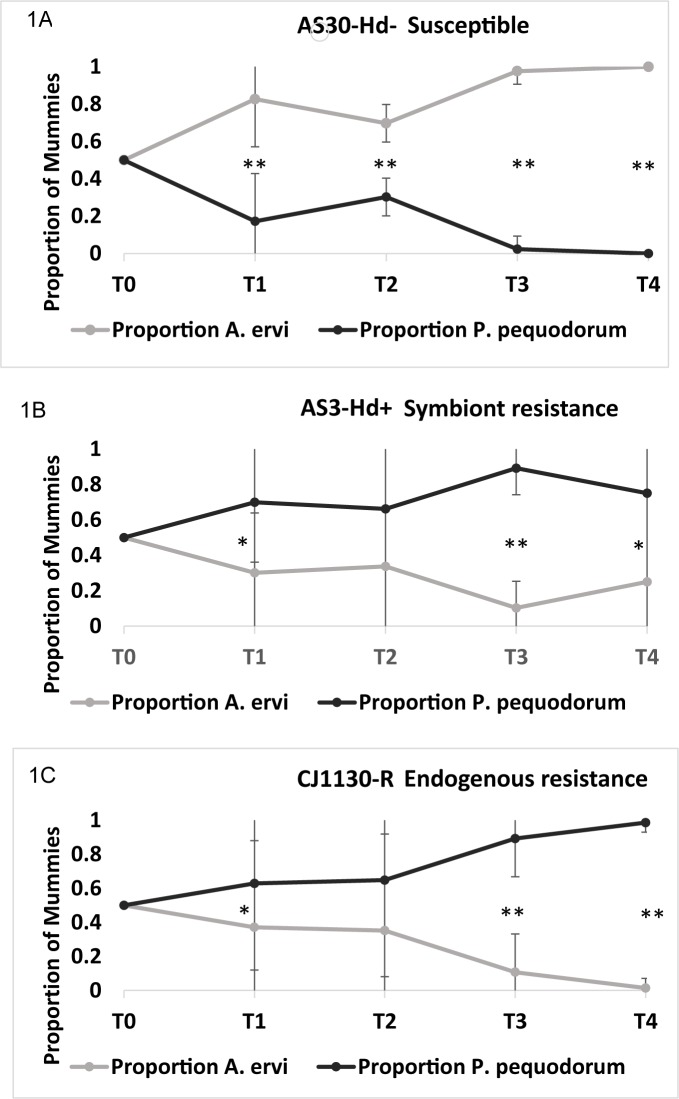
Competition assays between rival parasitoids in cages varying in amount and mode of resistance. (A) Line AS30-Hd: aphids susceptible to attack by wasp *A*. *ervi*. (B) Line S3-Hd+: Aphids with symbiont-based resistance. (C) Line CJ1130-R, Aphids with endogenous resistance. Graphs show mean proportion of mummies at each time point sampled. Bars represent range; * P < 0.05, ** P < 0.001.

In the population cage containing only susceptible aphids (AS30-Hd-), *A*. *ervi* outcompeted its rival *P*. *pequodorum* wasps and competitively excluded this wasp after 4 bouts of parasitism (Experiment 2A: Fig 1A, S1 Table). In the cages with symbiont- (AS3-Hd+) and aphid-based (CJ1130-R) resistance, however, we find that *P*. *pequodorum* outcompetes *A*. *ervi* in the majority of cases (Fig 1B and 1C, S1 Table) Cages with either mode of defense produced fewer mummies, and cages with interspecific competition produced fewer mummies. One of the four AS30-Hd- replicates (R4) was removed from this study because the wasps emerging from T1 caused high mortality to aphids in the subsequent bout of parasitism, likely due to multiparasitism and superparasitism, causing a crash in the parasitoid population.

**Fig 2:**
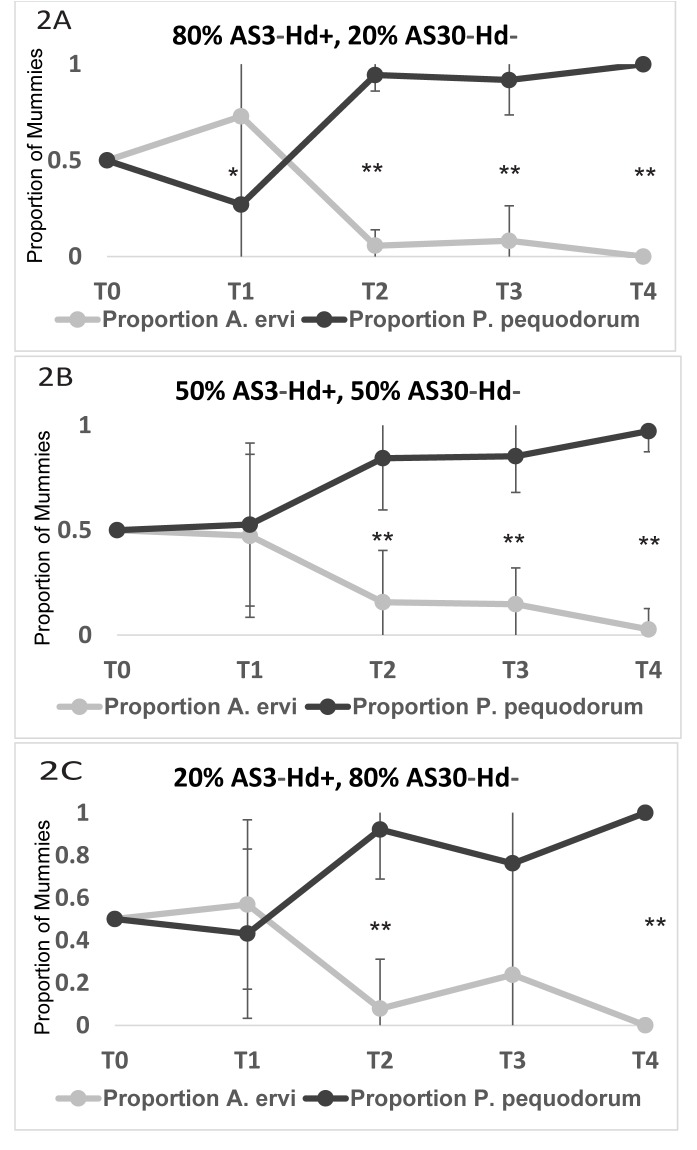
Competition assays between rival parasitoids in cages varying in proportion of aphids with symbiont-based defense. (A) 80% AS3-Hd+ (symbiont-based resistance)/ 20% AS30-Hd- (susceptible). (B) 50% AS3-Hd+/ 50% AS30-Hd-. (C) 20% AS3-Hd+, 80% AS30-Hd-. Graphs show mean proportion of mummies at each time point sampled. Bars represent range; * P < 0.05, ** P < 0.001.

We conducted a second population cage to determine what percentage of symbiont-based resistance in a population could affect the competition among wasps (Experiment 2B). Surprisingly, we find that *P*. *pequodorum* outcompetes *A*. *ervi* in all cages, even when the percentage of resistant aphids is only 20%, suggesting that even low levels of resistance specific to *A*. *ervi* in pea aphid populations can result in the persistence of *P*. *pequodorum* (Fig 2, S2 Table).

**Fig 3:**
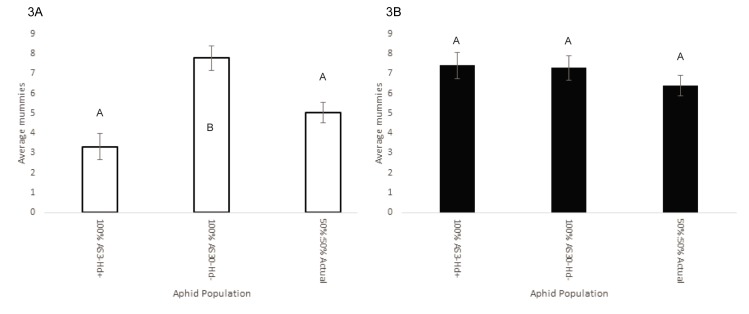
Mummy production (± SE) of *A*. *ervi* (3A) and *P*. *pequodorum* (3B) in the non-competitive parasitism assay. Letters indicate significant differences as determined by ANOVA and Tukey’s HSD post-hoc comparisons. The sets of letters are distinct for each wasp species.

In our small arena, non-competitive parasitism assays (Experiment 4), we found that the number of mummies produced varied according to the resistance composition of the aphid population and parasitoid species (*F*_2,154_ = 9.84, *P* < 0.0001) (Table 2). *A*. *ervi* produced significantly more mummies when attacking 100% AS30-Hd- aphids than 100% AS3 Hd+ aphids (Tukey’s HSD: *P*<0.0001) or the 50:50 mix (Tukey’s HSD: *P* = 0.0011) (Fig 3). The number of mummies produced by *P*. *pequodorum* did not significantly differ between the different aphid cohorts (Fig 3). While there were significant differences between the experimental blocks, these differences did not interact with the differences between the parasitoid species or the aphid cohorts.

**Table 2:** ANOVA table of mummies produced in the non-competitive parasitism assays. * indicate significant effects.

Source of Variation	Degrees of Freedom	Sum of Squares	Mean Square	F	P
Experimental Block	3	70.26	23.42	2.95	0.034*
Aphid resistance composition	2	110.01	55.01	6.94	0.0013*
Parasitoid species	1	87.14	87.14	10.99	0.0011*
Experimental Block*Aphid resistance composition	6	98.04	16.34	2.06	0.061
Experimental Block*Parasitoid species	3	43.67	14.56	1.84	0.14
Aphid resistance composition*Parasitoid species	2	156.03	78.01	9.84	0.000095*
Experimental Block*Aphid resistance composition* Parasitoid species	6	48.23	8.04	1.01	0.42
Residuals	154	1220.85	7.93		

**Fig 4:**
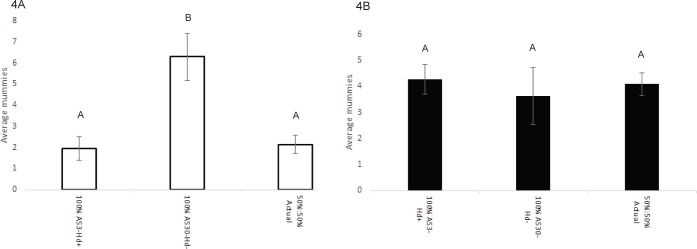
Mummy production (± SE) in the small-scale competitive cage assays. (A) Mummies produced by *Aphidius ervi*. (B) Mummies produces by *Praon pequodorum*. Letters indicate significant differences as determined by ANOVA and Tukey’s HSD post-hoc comparisons. The sets of letters are distinct for each wasp species.

In our small arena competition parasitism assays (Experiment 3), we found that *A*. *ervi* mummy counts varied significantly according to aphid treatment (*F*_2,65_ = 10.64, *P* = 0.00010) (Fig 4A) with a significantly larger number of mummies produced in 100% AS30-Hd- aphids than in either the 100% AS3-Hd+ aphids (Tukey’s HSD: *P* = 0.00015) and the 50:50 mix of the two aphid lines (Tukey’s HSD: *P* = 0.0029). *P*. *pequodorum* mummy counts did not differ among aphid lines (*F*_2,65_ = 0.52, *P* = 0.60) (Fig 4B. As with the larger scale cages, *A*. *ervi* produced more mummies than *P*. *pequodorum* only in arenas with 100% susceptible aphids, while any amount of resistance resulted in *P*. *pequodorum* producing more offspring.

We found no differences in *A*. *ervi* behavioral responses based on the presence of competitors, nor aphid lines varying in resistance (Experiment 5). The amount of time *A*. *ervi* spent walking, resting, grooming, or feeding did not differ between treatments (MANOVA: Pillai’s trace = 0.29, *F*_5,55_ = 0.85, *P* = 0.65), nor the number of aphids that were parasitized, escaped, or rejected did not differ between treatments (MANOVA: Pillai’s trace = 0.40, *F*_5,55_ = 1.71, *P* = 0.053). The number of interactions between *A*. *ervi* and *P*. *pequodorum* in which *P*. *pequodorum* was aggressive toward, retreated from, or ignored *A*. *ervi* was also unaffected by treatment (MANOVA: Pillai’s trace = 0.11, *F*_*2*,*27*_ = 0.50, *P* = 0.80). Finally, we found no differences in the number of *A*. *ervi* mummies that formed in response to each aphid cohort composition (*F*_5,55_ = 0.91, *P* = 0.48), which may be an artifact of the generally low and highly variable number of mummies produced with only one hour of foraging.

## Discussion

In North American pea aphid field populations, *A*. *ervi* is the dominant parasitoid, having competitively displaced numerous native and introduced parasitoids other than *P*. *pequodorum*, which remains at low levels in most sampled fields [38]. Using population cages, we show that both symbiont-based and endogenous aphid resistance traits can impact competition between these two rival parasitoids allowing for the persistence of *P*. *pequodorum*. In cages containing only aphids susceptible to attack by *A*. *ervi*, this wasp outcompetes and displaces *P*. *pequodorum*. However, in cages containing any proportion of aphids infected with the protective symbiont *H*. *defensa* (from 20 to 100%), or those with 100% endogenous resistance, *P*. *pequodorum* maintained viable populations for multiple generations and even displaces *A*. *ervi* in most instances (Figs 1 and 2). The finding that symbiont-resistance favors *P*. *pequodorum* was recapitulated in smaller, better replicated arenas (Fig 4). Thus, symbiont and host resistance traits likely provide a reservoir of susceptible hosts available only to *P*. *pequodorum* allowing this wasp to remain in natural populations when other species have been displaced. Consistent with this hypothesis, we found that *P*. *pequodorum* does equally well on all aphid lines in our no-competition assays, while *A*. *ervi* performance varies depending on the proportion of aphids with symbiont-based resistance (Fig 3). Such target-specific traits may impact community structure by differentially affecting species at higher trophic levels contributing to community richness and evenness.

It was surprising that even in cages (Experiment 2) with the lowest proportion (20%) of symbiont-resistant aphids, *P*. *pequodorum* outcompeted *A*. *ervi*. While *H*. *defensa* is found at varying frequencies in the field, most sampled populations exhibit > 20% *H*. *defensa* [37] indicating that relatively low proportions of resistant aphids may be sufficient for *P*. *pequodorum* persistence in the field. Given that *A*. *ervi* is a superior external competitor, the experimental design using closed cages with no migration and a limited number of hosts (ca. 800–1100 aphids in the larger-scale experiment and 20 or 30 aphids in the small-scale and behavior experiments) may have biased our experiments in favor of *P*. *pequodorum*. Larger cages with greater aphid abundances and lower overall parasitism rates may favor *A*. *ervi* relative to *P*. *pequodorum*, because the superior host-finding ability and faster attack rates of *A*. *ervi* gives it a greater intrinsic rate of increase than *P*. *pequodorum* [38, 48]. We found no difference in *A*. *ervi* foraging behavior due to competitors or aphid resistance traits (Experiment 5). In addition to aphid resistance traits and foraging abilities, other factors may directly or indirectly impact *P*. *pequodorum* abundance in field populations, including the availability of alternative aphid hosts [42] or differential resistance to particular parasitoid species occurring on different aphid food plants [14, 49].

The results from our small-scale competition assay (Experiment 3) were largely consistent with our larger cages, showing that defensive symbionts can alter the outcome of competitive interactions between rival wasps as host resistance traits and the presence of a competitor decreased *A*. *ervi*’s parasitism potential (Fig 4). In both competition (Experiment 3) and no-competition experiments (Experiment 4), we found that there was no effect of aphid cohort compositions for *P*. *pequodorum* (Figs 3 and 4). However, *A*. *ervi* produced significantly fewer mummies on the 50:50 mix and 100% AS3-Hd+ than on the 100% AS30-Hd-. If the combination of competition and resistance in some portion of the host population causes reductions in successful parasitism by *A*. *ervi*, then this may explain how *P*. *pequodorum* consistently outcompeted *A*. *ervi*, even at relatively low proportions of resistant hosts, in our larger cage studies.

While *P*. *pequodorum* consistently outperformed *A*. *ervi* in the presence of aphid resistance traits specific to *A*. *ervi*, we note an interesting anomaly in one cage replicate (Experiment 2). In the final sampling point (T4) of the four replicate cages containing 100% aphids with symbiont resistance (line AS3-Hd+; Fig 1B) *A*. *ervi* excluded *P*. *pequodorum* in one of the four replicate cages (*P*. *pequodorum* excluded *A*. *ervi* in the other three). This result is perplexing because at the penultimate sampling point (T3) *P*. *pequodorum* produced 89% of the mummies indicating a rapid and complete turnaround following just a single bout of parasitism.

Loss of *H*. *defensa* or APSE can lead to the instant loss of resistance to *A*. *ervi* [21] so we verified using diagnostic PCR that a sample of aphids (N = 8) screened shortly after this time point were all infected with both *H*. *defensa* and APSE. Of course, it is unlikely that symbiont or APSE loss among a subset of the population would have resulted in such a rapid and total change in competition outcomes. Another possibility is that the rapid evolution of counter-resistance specific to symbiont-based defense contributed to *A*. *ervi*’s resurgence, which could occur given that the same individual wasps emerging from prior parasitism bouts were reintroduced into the same replicate cage each round. While parasitoids, including *A*. *ervi*, have been shown to rapidly evolve counter-resistance specific to symbiont-based defenses [47, 50, 51], this mechanism also appears insufficient to explain the rapid turnover observed. It is also possible that some unknown factor, such as disease, contributed to the rapid decline in *P*. *pequodorum*’s performance in this single cage. Follow up studies are needed to determine whether *A*. *ervi* wasps can rapidly evolve the ability to overcome symbiont-based resistance, and if so, examining of mechanisms underlying increased wasp counter-resistance.

*P*. *pequodorum* is a superior internal competitor in cases of multiparasitism with *A*. *smithi* [52], and although no peer-reviewed studies are available, prior reports suggested that *P*. *pequodorum* is a superior internal competitor in cases of multiparasitism with *A*. *ervi* [40, 41]. However, it is possible that aphid- and symbiont-based resistance traits, rather than properties of the interacting wasps, mediate the outcome of multiparasitism events, which potentially contributed to our findings that *P*. *pequodorum* outperformed *A*. *ervi* in population cages containing resistant aphids. Instead, we found that *P*. *pequodorum* successfully outcompetes *A*. *ervi* in the multiparasitism assay (Experiment 1; Fig 1) all lines, including the uninfected, susceptible aphid line, confirming that *P*. *pequodorum* is indeed the superior internal competitor. That *P*. *pequodorum* does equally well in all lines indicates that neither symbiont nor aphid-based resistance mediates internal competition between these two wasp species. This finding also indicates that successful internal competitive ability was not likely an important factor contributing to *P*. *pequodorum*’s success in the population cages as this species was eventually excluded in treatments containing only highly susceptible aphids (Fig 1A), and the same aphid densities were used among all treatments. Our finding differs from a recent report that found that a strain of *H*. *defensa* infecting European pea aphids did change the outcome of internal parasitism between *A*. *ervi* and the chalcid wasp *Aphelinus abdominalis* [18]. In this instance, *H*. *defensa* has a large negative impact on *A*. *abdominalis* compared to *A*. *ervi*.

Pea aphid resistance traits specific to *A*. *ervi* may also indirectly improve *P*. *pequodorum*’s success in field populations. For example, pea aphids show fewer defensive behaviors, such as kicking parasitoids or dropping from plants, when infected with *H*. *defensa* [53, 54], which may increase *P*. *pequodorum’s* foraging success. However, in our no competition assays (Experiment 4), we did not find that *P*. *pequodorum* produced more mummies when attacking *H*. *defensa* infected aphids (Fig 3) compared to uninfected aphids. We used only a single aphid genotype and symbiont strain held in simple arenas with a single species (*Vicia faba*) of host plant, thus indirect effects may be stronger or weaker in field settings with other host plants or other genotypes. Nonetheless, symbiont-based resistance traits targeting one enemy may have both direct and indirect effects on non-targets.

## Conclusions

Here we show that aphid resistance traits from both endogenous and symbiont-based sources can influence competition between rival parasitoids, even when found at low percentages (20%) within populations. This result likely contributes to the persistence of *P*. *pequodorum* in field populations after the establishment of *A*. *ervi*, which displaced other parasitoids [38]. Thus, bacterial symbionts can play roles in influencing the composition of natural enemies attacking their hosts. Moreover, defensive symbionts and other resistance traits are may be more likely to target co-evolved enemies (but see [55]), and thus be less likely to harm native parasitoids that move onto the introduced host.

Complex aphid genotypes, comprising both aphid- and symbiont-encoded resistance to specific primary parasitoids, may influence the diversity and richness of the primary parasitoid community with effects that extend to other members within the food web, including other mutualists and other natural enemies. In turn, changing compositions of higher-order enemies and primary parasitoids can exert pressure leading to the maintenance of target specific resistance traits, allowing for more diversity in the food web.

## Supporting information

S1 Table Experiment 2A details. Successful parasitism as indicated by the number of mummies at each time point (T1 –T4; each corresponding to a bout of parasitism) for each replicate among population cages varying in modes (symbiont vs endogenous) and levels of resistance. * indicates that mummy production at that time differs significantly from that at time zero (T0) as determined by Fisher’s Exact Test. Ae = *A*. *ervi*; Pq = *P*. *pequodorum*.
(PDF)

S2 Table Experiment 2B details. Successful parasitism as indicated by the number of mummies at each time point (T1 –T4; each corresponding to a bout of parasitism) for each replicate among population cages varying in proportion of aphids with symbiont resistance. * indicates that mummy production at that time differs significantly from that at time zero (T0) as determined by Fisher’s Exact Test. Ae = *A*. *ervi*; Pq = *P*. *pequodorum*.
(PDF)
