# Correction: Aphid symbionts and endogenous resistance traits mediate competition between rival parasitoids

**DOI:** 10.1371/journal.pone.0190763

**Published:** 2018-01-02

**Authors:** Laura J. Kraft, James Kopco, Jason P. Harmon, Kerry M. Oliver

In the Funding section, the grant number from the National Science Foundation Dimensions in Biodiversity awards for KMO is listed incorrectly. The correct grant number is: 1240892.
