# Supplementary material for: Aphid symbionts and endogenous resistance traits mediate competition between rival parasitoids
Source: PLoS One. 2017 Jul 10;12(7):e0180729. doi: 10.1371/journal.pone.0180729 (PMC5507255; doi:10.1371/journal.pone.0180729)
Supplement: S1 Table — Successful parasitism as indicated by the number of mummies at each time point (T1 –T4; each corresponding to a bout of parasitism) for each replicate among population cages varying in modes (symbiont vs endogenous) and levels of resistance. * indicates that mummy production at that time differs significantly from that at time zero (T0) as determined by Fisher’s Exact Test. Ae = A. ervi; Pq = P. pequodorum. (PDF) [file pone.0180729.s001.pdf]

S1 Table. Successful parasitism as indicated by the number of mummies at each time point (T1 – T4; each corresponding to a bout of parasitism) for each replicate among population cages varying in modes (symbiont vs endogenous) and levels of resistance. * indicates that mummy production at that time differs significantly from that at time zero (T0) as determined by Fisher's Exact Test. Ae = *A. ervi*; Pq =*P. pequodorum*.

	**T1**	**T1**		**T2**	**T2**		**T3**	**T3**		**T4**	**T4**
Replicates	Ae	Pq	total wasps; mean/cage	Ae	P	total wasps; mean/cage	Ae	Pq	total wasps; mean/cage	Ae	Pq
AS30 (no *H. defensa*, susceptible genotype)											
AS30-1	143*	33*	**176**	128*	5*	**133**	96*	0*	**96**	n/a	n/a
AS30-2	53*	22*	**75**	114*	1*	**115**	143*	0*	**143**	n/a	n/a
AS30-3	101*	4*	**105**	56*	9*	**65**	93*	7*	**100**	710*	0*
total wasps; mean/cage	**297; 99**	**59; 20**	**356; 119**	**298; 99**	**15; 5**	**313; 104**	**332; 111**	**7; 2**	**339; 113**	710; 710	0
AS3 (susceptible genotype infected with protective APSE3 *H. defensa*)											
AS3-1	18*	74*	**92**	3*	18*	**21**	0*	40*	**40**	n/a	n/a
AS3-2	4*	28*	**32**	1	10	**11**	1*	43*	**44**	n/a	n/a
AS3-3	30	57	**87**	29*	8*	**37**	9*	43*	**52**	15*	0*
AS3-4	44	51	**95**	4	8	**12**	1	11	**12**	n/a	n/a
total wasps; mean/cage	**96; 24**	**210; 53**	**306; 77**	**37; 9**	**44; 11**	**81;20**	**11; 3**	**137; 34**	**148; 37**	15; 15	0
CJ1130 (no *H. defensa* but endogenously resistant genotype)											
CJ1130-1	2*	20*	**22**	2*	24*	**26**	3*	75*	**78**	0*	154*
CJ1130-2	6	8	**14**	29	16	**45**	10	28	**38**	17*	284*
CJ1130-3	11	16	**27**	32*	64*	**96**	1*	19*	**20**	n/a	n/a
CJ1130-4	3	2	**5**	5	10	**15**	1*	12*	**13**	n/a	n/a
total wasps; mean/cage	**22; 4**	**46; 12**	**68; 17**	**68; 17**	**114; 29**	**182; 46**	**15; 4**	**134; 34**	**149; 37**	**17; 9**	**438; 219**
