# Supplementary material for: Aphid symbionts and endogenous resistance traits mediate competition between rival parasitoids
Source: PLoS One. 2017 Jul 10;12(7):e0180729. doi: 10.1371/journal.pone.0180729 (PMC5507255; doi:10.1371/journal.pone.0180729)
Supplement: S2 Table — Successful parasitism as indicated by the number of mummies at each time point (T1 –T4; each corresponding to a bout of parasitism) for each replicate among population cages varying in proportion of aphids with symbiont resistance. * indicates that mummy production at that time differs significantly from that at time zero (T0) as determined by Fisher’s Exact Test. Ae = A. ervi; Pq = P. pequodorum. (PDF) [file pone.0180729.s002.pdf]

S2 Table. Successful parasitism as indicated by the number of mummies at each time point (T1 – T4; each corresponding to a bout of parasitism) for each replicate among population cages varying in proportion of aphids with symbiont resistance. * indicates that mummy production at that time differs significantly from that at time zero (T0) as determined by Fisher's Exact Test. Ae = *A. ervi*; Pq =*P. pequodorum*.

	T1	T1		T2	T2		T3	T3		T4	T4
Replicates	Ae	Pq	total wasps; mean/cage	Ae	Pq	total wasps; mean/cage	Ae	Pq	total wasps; mean/cage	Ae	Pq
80% AS3 Hd+, 20% AS30 Hd- (Majority infected with protective APSE3 *H. defensa*)											
80/20 1	147*	69*	**216**	5*	36*	**41**	18*	74*	**92**	0*	28*
80/20 2	64	63	**127**	3*	60*	**63**	1*	70*	**71**	n/a	n/a
80/20 3	73*	5*	**78**	9*	172*	**181**	0*	16*	**16**	n/a	n/a
80/20 4	107*	8*	**115**	2*	49*	**51**	0*	53*	**53**	n/a	n/a
total wasps; mean/cage	**391; 98**	**145; 36**	**536; 134**	**19; 5**	**317; 79**	**336; 84**	**19; 5**	**213; 53**	**232; 58**	**0; 0**	**28; 14**
50% AS3 Hd+, 50% AS30 Hd- (Equal proportion susceptible and infected with APSE3 *H. defensa*)											
50/50 1	79*	40*	**119**	14*	37*	**51**	1*	16*	**17**	n/a	n/a
50/50 2	77	91	**168**	3*	67*	**70**	19*	63*	**82**	1*	191*
50/50 3	19*	50*	**69**	31*	98*	**129**	8*	51*	**59**	9*	82*
50/50 4	28	45	**73**	2*	70*	**72**	13*	108*	**121**	0*	214*
total wasps; mean/cage	**203; 51**	**226; 57**	**429; 107**	**50; 13**	**272; 68**	**322; 81**	**41; 10**	**238; 60**	**279; 70**	**10; 5**	**487; 162**
20% AS3 Hd+, 80% AS30 Hd- (Majority susceptible)											
20/80 1	46*	91*	**137**	2*	47*	**49**	11*	80*	**91**	1*	78*
20/80 2	21	45	**66**	12*	37*	**49**	12*	45*	**57**	0*	16*
20/80 3	126*	80*	**206**	1*	81*	**82**	n/a	n/a		n/a	n/a
20/80 4	192*	76*	**268**	8*	104*	**112**	17	31	**48**	0*	18*
total wasps; mean/cage	**385; 96**	**292; 73**	**677; 169**	**23; 6**	**269; 67**	**292; 73**	**40; 10**	**156; 39**	**196; 49**	**1; 1**	**112; 37**
